# Product Development Partnerships: Case studies of a new mechanism for health technology innovation

**DOI:** 10.1186/1478-4505-9-33

**Published:** 2011-08-26

**Authors:** Richard T Mahoney

**Affiliations:** 1 Dengue Vaccine Initiative International Vaccine Institute Seoul, Korea

## Abstract

There is a continuing need for new health technologies to address the disease burdens of developing countries. In the last decade Product Development Partnerships (PDP) have emerged that are making important contributions to the development of these technologies. PDPs are a form of public private partnerships that focus on health technology development. PDPs reflect the current phase in the history of health technology development: the Era of Partnerships, in which the public and private sectors have found productive ways to collaborate. Successful innovation depends on addressing six determinants of innovation. We examine four case studies of PDPs and show how they have addressed the six determinants to achieve success.

## Introduction

Developing countries face many challenges to their social and economic development. With limited resources, they need to provide education to their children, ensure an adequate supply of food, stimulate the development of industry, build up an efficient transportation system, and provide health care to the population, among many others. To be successful in addressing these challenges, developing countries must be able to harness new technologies that are rapidly becoming available. In recent years, there have been rapid advances in the development and availability of new health technologies especially new vaccines and drugs for diseases that exact a heavy burden on developing countries. These include new vaccines against diarrhea, respiratory infections, and cervical cancer, and drugs such as the highly active antiretroviral therapies (HAART) for AIDS, and drugs for malaria. The availability of these new technologies holds great promise for addressing important diseases in developing countries, but also presents great challenges in financing and delivering these technologies to people in need.

Despite these recent advances in health technology there remain many diseases for which there are inadequate or no technologies that can reduce their burden on health. There are pressing needs for vaccines against AIDS, malaria, and tuberculosis, and against neglected tropical diseases (NTDs) such as hookworm, schistosomiasis, and dengue. There is also a need for new and improved drugs against most of these diseases [1].

These twin challenges of financing and delivering existing technologies and developing new and improved technologies have led to important initiatives by developing countries and by the global health community.

The Global Fund to Fight AIDS, Tuberculosis and Malaria has been established to mobilize donor funds to procure and supply needed drugs for developing countries [2]. The GAVI Alliance similarly has been established to procure and supply needed vaccines for developing countries [3]. Both have been able to raise billions of dollars on an annual basis for these important undertakings. Not only have they seen the need to finance the procurement of these technologies but they have also increasingly appreciated the challenges of delivering the technologies in developing countries. They have been allocating increasing funds to support health systems development.

With respect to the development of new and improved technologies, a number of additional new initiatives have been launched. These initiatives are referred to as product development partnerships (PDPs) that address a wide range of diseases and work on accelerating the development of drugs, vaccines, diagnostics and other technologies [1]. The single largest donor for the PDPs is the Bill & Melinda Gates Foundation. Support has also been provided by the World Bank, other foundations, and bilateral donors. The PDPs have, in common, a strategy of promoting collaboration between public and private sector institutions in developed and developing countries. The PDPs are nonprofit entities operating with philanthropic funds and form collaborative partnerships with private sector health technology corporations to design and implement product development programs for specific health technologies. For example, the PATH Malaria Vaccine Initiative has been working closely with GlaxoSmithKline Biologicals in the development of a malaria vaccine which is now in Phase 3 trials in African countries. The Global Alliance for TB Drug Development (TB Alliance) has been working with a number of companies in the development of new drugs against tuberculosis. The Pediatric Dengue Vaccine Initiative of the International Vaccine Institute has been working with a number of companies to promote the development of five candidate vaccines against dengue.

The launch of these various PDPs is a key part of the current era of health technology innovation which we refer to as the Era of Partnerships [4]. The Era of Partnerships is the fourth in a series of Eras in modern health technology innovation reaching back to the 1850s. Each of these Eras has been characterized by different levels of engagement and investment by the public and private sectors.

In the Era of the Public Sector (1850 to 1915), health technology innovation was dominated by public sector institutions primarily in Europe and epitomized by the work of Louis Pasteur and the Pasteur Institute to develop and disseminate vaccines around the world. The Era of the Private Sector (1915 to 1970) was launched by the realization of European - predominantly German - chemical companies that there was a highly profitable market for drugs. This led to a rapidly increasing level of investment in the pharmaceutical industry including research, development, manufacture, and marketing. This Era led to the development and global distribution of a large number of important technologies that revolutionized health care. However, with the end of the Second World War, and led by the United States, a number of developed country governments and foundations became concerned that the health technology revolution had largely bypassed the poor in developing countries. Not only were the new technologies not reaching the poor in developing countries, but technologies that were needed by people in developing countries were not being developed. This realization led to the Era of Public Sector Reawakening (1970 to 2000) and involved support for efforts to develop new contraceptives at the Population Council in New York City and the Human Research Programme (HRP) at WHO in Geneva, and a variety of technologies at the Tropical Disease Research Program (WHO), and PATH, a 501(c)3 non-profit in Seattle, Washington. Not only did these programs succeed in developing several new technologies that were introduced into developing countries but they provided an essential and invaluable means for designing and testing strategies by which the public sector could stimulate the development of health technologies for use in developing countries. One of the foremost lessons learned from this experience was that close collaboration between the public and private sector was essential for success. From the perspective of the second decade of the 21st century, this lesson may seem obvious. But in the early 1970s, there was a huge gulf between the public and private sectors about health technology innovation. The public sector characterized the private sector as largely unsympathetic to the needs of the poor and especially the poor in developing countries. The private sector characterized the public sector as being largely incapable of developing new health technologies and with little understanding of how to develop, manufacture, and market health technologies in a sustainable manner.

But, based on the new body of knowledge from the operation of the various public sector product development groups, and largely funded by the Bill & Melinda Gates Foundation, the Era of Partnerships was launched around the year 2000. This Era is characterized by an accommodation between the public and private sectors in which each sees that the other has something to contribute to their individual and collective goals. It has involved the establishment of a large number of PDPs including for vaccines against malaria, HIV, tuberculosis (TB), hookworm, meningitis, cholera, dengue, and pneumococcal disease, and for drugs against malaria, TB, HIV infection, visceral leishmaniasis, and technologies such as diagnostics. Each of these PDPs forms strong partnerships with private companies that are governed by formal agreements dealing with issues including intellectual property, regulatory pathways, markets, manufacture, and price. The objective of these agreements is to lay out rules of the collaboration that meet the differing and complementary goals of the partners. The PDP wants a safe, effective and affordable product available to the poor in developing countries. The company wants a safe, effective and affordable product that provides a robust return on investment. During the last decade, an increasingly formal system of exchange of information and management know how has evolved among the PDPs that has allowed them to benefit from knowledge of what works and what doesn't work.

In this paper we seek to summarize some of the lessons that have been learned about how PDPs and private sector pharmaceutical firms can effectively collaborate to promote innovation in global health. We use the case study methodology.

## Methods

This work was conducted by examination of publicly available documents and by interviews with key individuals at each of the organizations which are the subject of this study. The case studies examined herein were selected on several criteria. The programs of all PDPs were reviewed through websites, annual reports and newsletters, and examples of successful projects were selected. A subset of those projects was selected where there was the availability of the most detailed documentation about the project. The final criterion was the availability of staff at the PDPs willing to discuss the projects in detail and provide important information.

## Results

We propose that there are six broad areas of collaboration - determinants of innovation - between public and private sector organizations to promote innovation in health technologies [5].

1. The design and execution of research and development programs from preclinical studies to licensure.

2. Analysis and planning for the marketing and distribution of new technologies in individual developing countries

3. Analysis and planning for the procurement and supply of new health technologies by the global health community

4. Planning and implementation of manufacturing capabilities

5. Establishment and implementation of regulatory systems to ensure safe and effective products

6. Establishment and implementation of intellectual property rights (IPR) management systems

These six determinants of innovation are comprehensive in that they span all activities that are necessary and sufficient for the development, and introduction of health technologies in developing countries. We examine several case studies that illustrate the need to address each of the determinants of innovation albeit in different proportions depending on the product.

### Medicines for Malaria Venture and Coartem^® ^Dispersible

The Medicines for Malaria Venture (MMV) is based in Geneva Switzerland and was founded in November 1999 with funding from the Rockefeller Foundation, the World Bank, and the governments of Switzerland, the United Kingdom and the Netherlands. Its expenditures on research and development grew from about $2.3 million in 2000 to an average of about $45 million from 2006 through 2009 [6]. MMV operates according to a standard R&D process with the following components: compound screening and hit-to-lead identification, lead optimization, preclinical development and candidate selection, clinical phase 1, clinical phase 2, clinical phase 3, and registration and launch. In 2006, MMV committed also to prioritize Access & Delivery work in ensuring the broad uptake and health impact of products that emerge from its drug development pipeline - this area of work focuses around enhancing the acceptance (internationally and nationally), uptake (particularly in rural access-deprived areas), and measurement of impact of MMV products in "real-life" settings.

MMV has collaborated with pharmaceutical companies in the screening and hit-to-lead identification component of the R&D process. For example, they have undertaken a screening initiative to harness private sector research capabilities to examine large libraries of small molecule compounds for effectiveness against the malaria parasite. Collaboration takes place in all other stages of the process. In addition to providing funding, MMV also provides substantial know how, technical and supervisory inputs by its staff and through an Expert Advisory Committee and also seeks in-kind contributions in the form of staff, laboratory space, equipment and operates with a zero overhead policy.

In collaboration with Novartis, MMV has developed and launched a combination treatment for malaria, Coartem^® ^Dispersible. This product is a sweet-tasting, dispersible pediatric dose formulation of a malaria drug combination treatment used widely for adults. It is highly effective and well accepted by children. The intellectual property including know-how belongs to Novartis for both the combined use of the active ingredients, artemether and lumefantrine, and for the dispersible formulation. The agreement between MMV and Novartis includes a commitment by Novartis to distribute the product in malaria-endemic countries. If Novartis fails to do so, MMV gets a sub-licensable license to manufacture and sell in those countries. Further Novartis agreed to make the product available at cost to the public sector in malaria-endemic countries [7]. To bring this product to developing countries, MMV sponsored clinical bridging studies that assessed the safety and efficacy of the new formulation in children [8] and allowed the registration of this new product. In December 2009, it was approved by Swissmedic, then went on to obtain WHO prequalification, and was placed on the WHO essential medicines list.

By early 2010, the product had been approved by 24 national regulatory agencies. MMV worked with Novartis to establish adequate production, to formulate extensive plans for marketing and distributing the product, and helped develop the regulatory pathway. MMV has also engaged country policymakers to encourage the revision of national treatment guidelines in favor of improved solutions for treating pediatric malaria. Lastly, MMV collaborates with Novartis in training health workers and supporting national initiatives to make Coartem Dispersible more widely available in rural areas through the promotion of community-healthcare-worker programming.

As of June 2010, over 35 million treatments have been supplied. In sum, MMV addressed all six determinants of innovation leading to a successful outcome. It supported product development, collaborated on production scale up, helped arrange regulatory approvals, entered into facilitating IP management arrangements, and helped developed international and national distribution systems.

### PATH and the Japanese encephalitis project

PATH was founded in the mid-1970s and has grown to be one of the world's largest non-profit technology organizations concerned with health in developing countries. One of its projects was to promote the introduction of Japanese encephalitis vaccine into Bangladesh and India [9]. Japanese encephalitis is the leading cause of viral encephalitis in Asia and is the leading neurological infection in that region. Throughout Asia there are 50,000 cases reported annually and it is believed that this number represents a severe underreporting. There are 10,000 to 15,000 deaths annually with a 5 percent to 35 percent case fatality rate. Of considerable importance with respect to Japanese encephalitis is the post-infection disability rate which ranges from 30 percent to 75 percent of cases and is primarily neurological. A vaccine against Japanese encephalitis has been available for many years but its use has been limited. This vaccine is produced using fetal mouse brains. Use of the vaccine has been limited because of inadequate supply due to the difficult production process, cost of production, a difficult administration schedule, and, probably most important, concern about side effects resulting from mouse brain material remaining in the vaccine. A less expensive and less reactogenic vaccine, referred to as SA-14-14-2, was developed in China and could be administered in a two-dose regimen over 2.5 months to achieve high levels of protection [10]. The PATH project had the goal of introducing this vaccine into Bangladesh and India.

The project in India had to overcome many barriers. First, there was not a full appreciation by policymakers in India of the importance of Japanese encephalitis as a disease. Therefore a surveillance program was undertaken that documented the significant incidence of Japanese encephalitis and its burden on the health system. Second, while medical professionals in India appreciated that the mouse brain vaccine was associated with certain side effects, they were not willing to accept the introduction of the Chinese vaccine because they were unsure about the rigor of the Chinese national regulatory authority. To address this barrier, PATH facilitated a rigorous review of all available data of clinical testing and evaluation of the Chinese vaccine. The WHO Global Advisory Committee on Vaccine Safety also carried out a review of the data for the SA-14-14-2 vaccine and acknowledged the excellent safety and efficacy profile while calling for some additional clinical studies of the vaccine. Another important event was the licensure by the Korean FDA of the Chinese vaccine (the KFDA has WHO approval). These reviews and licensure led to an acceptance by Indian authorities (National Technical Advisory Group on Immunization and the national regulatory agency) of the Chinese vaccine. Another issue with respect to supply is the policy of India to rely on domestic manufacturers for supply of its vaccines. Indian authorities wished to have the know-how for production of the vaccine transferred to India, but the Chinese authorities did not agree to this request. Eventually the Indian decision-makers accepted the situation on the condition that a local Indian government company (Hindustan Latex Limited) be the agent for the Chinese manufacturer. Once Indian authorities accepted the idea of introducing the vaccine, there were a number of activities that needed to be completed including the preparation of an introduction plan, operational guidelines, training materials, and vaccine procurement. It was also necessary to set up a monitoring and evaluation plan including monitoring of adverse events following immunization. Finally clinical studies had to be designed and implemented to measure immunogenicity, safety and viremia [11].

In sum, PATH addressed all six determinants of innovation. The key issues in the introduction of an improved Japanese encephalitis vaccine into India involved establishing a domestic market, i.e. a vaccination program, resolving numerous regulatory issues, and establishing an international supply mechanism of the vaccine from China to India. Manufacturing issues were addressed through interactions with the Chinese producer. Finally, IP issues were important to the extent that they involved the unwillingness of the Chinese manufacturer to transfer the production technology to India, but this matter was successfully addressed.

### The International Vaccine Institute and the Cholera Vaccine Initiative (CHOVI)

The International Vaccine Institute (IVI) was founded in Seoul, Korea in 1997 as an autonomous international organization under the Vienna Convention. The treaty establishing the IVI has 40 country signatories plus WHO. It is the only international organization committed to the development of new vaccines for people in developing countries. It has managed several PDPs including ones for cholera, shigellosis, and salmonella (DOMI - Diseases of the Most Impoverished), cholera (CHOVI - Cholera Vaccine Initiative), typhoid (VIVA - Vi Typhoid Vaccine Program), and for dengue (PDVI - Pediatric Dengue Vaccine Initiative - see below). The cholera program which grew out of the DOMI program has successfully addressed the need for highly qualified vaccine production facilities in developing countries.

The cholera case study [12] is an important illustration of the links among the determinants of innovation. Establishing manufacturing facilities has to take into account both market (domestic and international) and regulatory considerations. As argued by Lall [13], in terms of innovation, a company's entry into international markets is fundamentally different from entry into domestic markets. Entering the international market in pharmaceuticals requires that the product meets certain regulatory standards which are often much more rigorous than those to be found in most developing countries. Countries importing vaccines produced in developing countries will often require that the vaccines be approved by one or more regulatory authorities of developed countries. Alternatively they may require that the vaccine obtain WHO pre-qualification. To provide pre-qualification, WHO sends teams of highly skilled individuals to assess the vaccine including the production facility and the national regulatory authority. The vaccine is pre-qualified only if both the production facility and the national regulatory authority meet certain requirements [14]. Additionally, cholera is a disease affected some of the poorest countries and these countries will have to rely on donors to obtain the vaccine. Often donors use UNICEF procurement services and UNICEF also required WHO prequalification.

Vietnam had established the production of a cholera vaccine with technology transfer from Sweden. There were certain problems in the Vietnamese facility with compliance with the WHO guidelines for the production of killed oral cholera vaccines and with the quality of the vaccine including residual cholera toxin. The IVI, mostly at its facilities in Seoul, worked with the Vietnamese facility and addressed these compliance issues and were able to improve the vaccine in several ways. The new vaccine formulation and new quality control assays were made available to the Vietnamese manufacturer and are now being used by that manufacturer. However it was now necessary to establish production in a country that had a WHO prequalified national regulatory authority. The IVI staff facilitated the transfer of the technology to Shantha Biotechnics in India which had been successful in obtaining WHO prequalification for other vaccines. Clinical trials of this new cholera vaccine were conducted in both Vietnam and India by the IVI with Vietnamese (NIHE) and Indian (NICED) collaborators, and the vaccine was found to be both safe and effective [15]. The vaccine has been licensed in both countries and is being introduced into immunization programs. Subsequent to this work, a controlling interest in Shantha was acquired by sanofi pasteur, a member of the sanofi aventis group, from bioMerieux.

The next step will be to export the vaccine to other developing countries. As noted above, this will require the producers to obtain some form of regulatory review satisfactory to the importing countries which will in most cases include WHO prequalification. This will be an important further step in the development of innovative capabilities of the producers and the relevant government agencies.

This case study illustrates how a PDP, focusing on the needs of the poor in developing countries, can be successful in improving the manufacturer of a needed vaccine and transferring the technology to multiple developing countries. Because cholera is a disease that occurs among only the poor in developing countries, it is certain that only the public sector would be willing to allocate the required resources to achieve this success. However the ultimate success of the program required the participation of a private sector manufacturing company in India.

It is important to note also that this cholera case study provides a very robust example of a PDP generating and employing extensive background data on the incidence, disease burden, and vaccine demand and willingness to pay, and then applying those data to support vaccine demonstration projects and vaccine cost effectiveness analyses [16-23]. This evidence provided a basis for a presentation to WHO SAGE which recommended use of the vaccine in late 2009 [24]. Finally, IVI has prepared a detailed Vaccine Investment Case to support the procurement and distribution of the vaccine in endemic countries. The comprehensive approach used by IVI illustrates how success can be ensured by a PDP addressing research, domestic and international markets, regulatory, and production issues in a highly integrated way.

An interesting aspect of this case study is that there were no patents that either facilitated or constrained the process. However the IVI and its collaborators developed important intellectual property rights in the form of know-how which are protected by confidentiality agreements. Thus, IP was important to the success of this project even in the absence of patents. This is the general case with respect to vaccines where know how is often more important than patents.

In sum, IVI addressed all determinants of innovations - issues of product development, scale up of manufacture, regulatory approvals, IP management, and development of national and international distribution systems. Work continues on the international distribution challenges.

### The International Vaccine Institute and the Pediatric Dengue Vaccine Initiative (PDVI)

Dengue is the world's most important vector-borne viral disease threatening over 3.6 billion people worldwide and resulting in more than 500,000 cases per annum. While mortality from the disease is low (about 23,000 deaths per year most among children), morbidity associated with dengue levies heavy burdens on developing countries for their health systems and for individual families who must pay the costs of treatment for this disease.

The PDVI was established in 2002 and has received funding from the Rockefeller Foundation and the Bill & Melinda Gates Foundation. Its programs have been designed specifically to address each of the six components of innovation [25]. It has invested in facilitating research and development on a number of new vaccines being sponsored by a variety of companies, both public and private. It has worked on developing a global demand and appreciation of the importance of dengue vaccine through advocacy activities. It has worked with individual countries to plan for the introduction of the new vaccines to their domestic markets. For both domestic and global markets, it carried out an extensive evaluation of the potential uptake of dengue vaccines in an initial five-year period following licensure [26]. The PDVI undertook an extensive assessment of the intellectual property rights situation with respect to dengue vaccines and concluded that there were no significant barriers to the development of the current portfolio of vaccines in advanced testing [unpublished data]. The PDVI, in close collaboration with the World Health Organization, has worked extensively on establishing a regulatory pathway for dengue vaccines [27]. A detailed discussion of the PDVI programs has been published [25]. Substantial progress has been achieved in dengue vaccine development with licensure of a first vaccine possible in 2014. In the next several years, attention will be directed towards developing country-specific introduction plans, establishing provisional vaccine strategies, clarifying the epidemiology and burden of disease of dengue, and ensuring adequate manufacturing facilities worldwide to supply the expected large demand for dengue vaccines. [In January 2011, the PDVI was renamed the Dengue Vaccine Initiative consisting of a consortium of four organizations: IVI, International Vaccine Access Center of Johns Hopkins Blumberg School of Public Health, the Initiative for Vaccine Research of WHO, and the Sabin Vaccine Institute.]

In sum, the PDVI has addressed the six determinants of innovation. It supported two companies to complete preclinical research including production scale up. It addressed issues of IP management, regulatory approvals, and development of national and international distribution systems. Work on the last two issues is a high priority for the continuing activities of DVI.

## Discussion

These case studies illustrate how PDPs address each of the six determinants of innovation (see Table 1). The relative extent to which they address each determinant varies according to a number of variables including the product, the target group of affected individuals, the countries into which the product will be introduced, and the willingness of industry to invest in product. Progress achieved varies on the stage of the product's development at the time the PDP initiates its work. Also the relative priority accorded to each determinant of innovation will differ depending on the stage of product development. For example, manufacture is much more important if a product is approaching licensure for distribution.

**Table 1:** PDP work on determinants of innovation

Case Study	R&D	National Markets	International Markets	Manufacture	Regulatory Systems	IP management
**MMV and Coartem Dispersible**	Sponsored clinical trials	Supports training of local health workers and expansion of use of product by new community health worker programs. Engages national policymakers to encourage policy shift in favor of improved child treatment and survival.	Ensure alignment of product with Global Fund, UNICEF and PMI requirements for international procurement. Collaborated with Novartis in international launch and communication activities around product introduction.	Worked with Novartis to plan for production volume	Helped back regulatory pathway for eventual approval in 24 countries. Facilitate country specific regulatory review in key countries in sub-Saharan Africa.	Obtained access commitments from Novartis which have ensured accessible/affordable pricing of product for public sector uptake.
**PATH and JE vaccine**	Sponsored surveillance research. Helped set up monitoring and evaluation plan.	Helped establish state-level distribution programs	Arranged international supply of vaccine from China	Obtained agreement for continued manufacture in China	Facilitated review by Indian authorities to obtain approval	Respected China producers rights and obtained agreement for supply at low price
**IVI and cholera vaccine**	Undertook clinical trials to evaluate safety and efficacy	Establishing an investment case for cholera vaccine	In progress	Developed new production and quality control methodologies and transferred to Vietnam and India	Obtained licensure in India and Vietnam	Developed know how which was protected to ensure control of product
**IVI and dengue vaccine**	Helped two biotech move product from lab to Phase 1 study	Undertook study of potential markets in most endemic countries	Initiated work with international agencies to raise awareness of dengue	Worked with developing country manufacture to establish production	Worked with WHO and national regulatory authorities to develop pathway	Undertook detailed freedom to operate study showing no serious constraints

Over the last decade, PDPs have become an increasingly important means to accelerate the development of health technologies for neglected diseases. By 2007, they obtained (omitting funding by the U.S. NIH which focuses on basic research) 42.0% of external global research and development funding for neglected diseases [28]. While in the early years there was uncertainty about whether PDPs could be successful [29,30], it is now generally accepted that they can be and have been effective [28].

However, they are a relatively new form of organization for health innovation. Before the mid-1990s, there were only a few non-profit organizations attempting to undertake product development in health. One of these was PATH which had been launched in the mid-1970s. The PATH experience has been summarized by Elias [31]. He describes a value chain of five stages. These five stages encompass the six determinants of innovation as we indicate by inserting the determinant numbers that are related to each stage. discovery and research (1, 6), development of discoveries into usable products (1, 4, 6), regulatory processes to ensure product safety and licensure (5), introduction of new technologies into health systems (2), and scale-up and effective use of products by populations (2, 3, 4).

The experience of PATH has been utilized by many PDPs, and they have also used other models such as biotechnology companies and product development arms of pharmaceutical companies. However, as noted by Elias, it is exactly in the areas of understanding of health systems in developing countries and introducing products into those health systems (what we refer to as analysis and planning for the marketing and distribution of new technologies in individual developing countries) where private industry often does not have in-depth experience. Thus, the PDPs have been engaged in creating, largely *ab initio*, a comprehensive system and methodology for product innovation while launching and implementing programs, i.e. they have been engaged in "learning by doing."

Of particular interest is the evolution of the understanding of the role of IP in health technology development and access. This is well illustrated by the work of the UK and WHO on IP. In the 1990s and early 2000s, it was often asserted that patents and other forms of IP were major factors limiting access by the poor to new health technologies. A Commission formed by the UK examined this issue in depth. The argument was well summarized in the Commission's report [32]:

On the one side, the developed world side, there exists a powerful lobby of those who believe that all IPRs are good for business, benefit the public at large and act as catalysts for technical progress. They believe and argue that, if IPRs are good, more IPRs must be better. On the other side, the developing world side, there exists a vociferous lobby of those who believe that IPRs are likely to cripple the development of local industry and technology, will harm the local population and benefit none but the developed world. They believe and argue that, if IPRs are bad, the fewer the better.

The Commission's report is comprehensive and complex. The report notes that research and development of new products and their eventual availability to the poor are affected by a wide range of factors.

The heart of the problem is the lack of market demand sufficient to induce the private sector to commit resources to R&D. Therefore, we believe that presence or absence of IP protection in developing countries is of at best secondary importance in generating incentives for research directed to diseases prevalent in developing countries.

Subsequent to the work of the UK Commission, WHO formed a Commission on Intellectual Property Rights, Innovation and Public Health. It similarly concluded [33]:

In successive phases of the innovation cycle - from fundamental research to the discovery, development and delivery of new products - the multiplicity of financial and other incentive mechanisms, and the scientific and institutional complexities of biomedical innovation have had to be considered. At each phase intellectual property rights may play a greater or lesser role in facilitating the innovation cycle. Other incentive and financing mechanisms to stimulate research and development of new products are equally necessary, along with complementary measures to promote access.

Thus, as argued in this paper, IP is important and it can affect access - both positively and negatively - but it is only one of six factors that affect innovation. And it is rarely the most important factor.

There are differences between PDPs that address drugs and those that address vaccines. Those that address drugs especially where they are working on reformulations of existing products or devising new production methods, as in the cases of MMV and TB Alliance, have sufficient resources to "own" the products, i.e. they pay for the development, testing and regulatory licensure. The costs for this kind of work are in the tens of millions of dollars. Under these circumstances they can have great control of plans for marketing, distribution and pricing. Those that address vaccines (the Meningitis Vaccine Program at PATH is an exception) rarely have the required funds to own the product and are much more reliant on the resources of private companies. The total cost of developing a new vaccine may well exceed $1 billion. Under these circumstances, the PDPs have less influence on development, marketing and distribution and other determinants. Nevertheless, both kinds of PDPs must address the six determinants of innovation.

Perhaps the most important development of the last decade is that PDPs have shown to be a critical and necessary component in ensuring access by the poor in developing countries to needed health technologies (Wells 2011). Recent moves by additional donors such as the Department for International Development of the United Kingdom, Ministry of Foreign Affairs of the Netherlands, and the Ministry of Foreign Affairs of Germany to allocate more money to PDPs indicate that this new organizational framework should be sustainable for years to come. A critical factor in achieving sustainability, as laid out in this paper and elsewhere [34], will be for the PDPs to address each of the six determinants of innovation. Success in doing so will be key to success in their work, which will be the strongest argument for sustained support.

This paper seeks to contribute to the development of a comprehensive system and methodology by bringing to bear the insights of innovations studies with the actual experience of PDPs.

## Competing interests

The authors declare that they have no competing interests.

## References

[B1] Moran M Guzman J Henderson K Ropars AL McDonald A McSherry L Wu L Omune B Illmer A Sturm T Zmudzki F Neglected disease research & development: new times, new trends 2009 The George Institute for International Health: Sydney 101

[B2] The Global Fund 2010: Innovation and Impact 2010 The Global Fund to Fight AIDS, Tuberculosis and Malaria: Geneva 132

[B3] GAVI Saving lives and protecting health: Results and opportunities 2010 GAVI Alliance: Geneva

[B4] Mahoney R Morel CM A Global Health Innovation System Innovation Strategy Today 2006 2 1 1 12

[B5] Mahoney R Lee K Yun MK The evolution of biotechnology in Korea: a Framework for Analysis; a case study of the vaccine industry Innovation Strategy Today 2005

[B6] Annual Report 2009. MMV: Medicines for Malaria Venture 2010 http://www.mmv.org/sites/default/files/uploads/docs/publications/annual_report_2009_0.pdf [cited 2010 May 21]

[B7] Fonteilles-Drabek S Intellectual Property Provision for Coartem Dispersable Personal Communication 2010 Geneva

[B8] Abdulla S Sagara I Borrmann S D'Alessandro U Gonzalez R Hamel M Ogutu B Martensson A Lyimo J Maiga H Sasi P Nahum A Bassat Q Juma E Otieno L Bjorkman A Beck HP Andriano K Cousin M Lefevre G Ubben D Premii Z Efficacy and safety of artemether-lumefantrine dispersible tablets compared with crushed commercial tablets in African infants and children with uncomplicated malaria: a randomised, single-blind, multicentre trial The Lancet 2008 372 9652 1819 1827 10.1016/S0140-6736(08)61492-0 18926569

[B9] Jacobson J The Burden of JE in Southeast Asia: The Role of NGOs in the Fight Against the Disease 2008 http://www.path.org/vaccineresources/files/Role_of_NGOs.ppt [cited 2010 MAY 24]

[B10] Tsai TF Yong-Xin Y JL Li Putvatana R Ran Z Shouqui W Halstead S Immunogenicity of live attenuated SA14-14-2 Japanese encephalitis vaccine--a comparison of 1- and 3-month immunization schedules J Infect Dis 1998 177 1 221 3 10.1086/517358 9419193

[B11] Jacobson J Introduction of Japanese Encephalitis Vaccine Asia Pacific Dengue Prevention Board: Vaccine Introduction and Implementation 2009 Siem Reap, Cambodia

[B12] DeRoeck D The IVI's innovative approach to closing the gap between vaccines for industrial and developing countries Global Forum Update on Research for Health 2008 5 Geneva: Global Forum for Health Research 138 142 Fostering innovation for global health.

[B13] Lall S Exports of Manufactures By Developing Countries: Emerging Patterns of trade and location Oxford Review of Economic Policy 1998 14 2 54 73 10.1093/oxrep/14.2.54

[B14] Milstien JB Kaddar M The role of emerging manufacturers in access to innovative vaccines of public health importance Vaccine 2010 28 9 2115 2121 10.1016/j.vaccine.2009.12.036 20044054

[B15] Sur D Lopez A Kanungo S Paisley A Manna B Ali M Niyogi SK Park JK Sarkar B Puri MK Kim DR Deen JL Holmgren J Carbis R Rao R Nguyen TV Donner A Ganguly NK Nair GB Bhattacharya SK Clemens JD Efficacy and safety of a modified killed-whole-cell oral cholera vaccine in India: an interim analysis of a cluster-randomised, double-blind, placebo-controlled trial Lancet 2009 374 9702 1694 702 10.1016/S0140-6736(09)61297-6 19819004

[B16] Thiem VD Deen JL von Seidlein L Canh G Anh DD Park JK Ali M Danovaro-Holliday MC Son ND Hoa NT Holmgren J Clemens JD Coverage and costs of mass immunization of an oral cholera vaccine in Vietnam J Health Popul Nutr 2003 21 4 304 8 15043004

[B17] Deen JL von Seidlein L Sur D Agtini M Lucas MES Lopez AL Kim DR Ali M Clemens JD The high burden of cholera in children: comparison of incidence from endemic areas in Asia and Africa PLoS Negl Trop Dis 2008 2 2 e173 10.1371/journal.pntd.0000173 18299707 PMC2254203

[B18] Agtini MD Soeharno R Lesmana M Punjabi NH Simanjuntak C Wangsasaptra F Nurdin D Pulungsi SP Rofiq A Santoso H Pujarwoto H Sjahrurachman A Sudarmono P von Seidlein L Deen JL Ali M Lee HJ KIm DR Han OP Park JK Suwandono A Ingerani Oyofo BA Campbell JR Beecham HJ Corwin AL Clemens JD The burden of diarrhoea, shigellosis, and cholera in North Jakarta, Indonesia: findings from 24 months surveillance BMC Infect Dis 2005 5 89 10.1186/1471-2334-5-89 16242013 PMC1276796

[B19] Sur D Deen JL Manna B Niyogi SK Deb AK Kanungo S Sarkar L Kim DR Danovaro-Holliday MC Holliday K Gupta BK Ali M von Seidlein L Clemens JD Bhattacharya SK The burden of cholera in the slums of Kolkata, India: data from a prospective, community based study Arch Dis Child 2005 90 11 1175 81 10.1136/adc.2004.071316 15964861 PMC1720149

[B20] Ali M Emch M Yunus M Sack D Lopez AL Holmgren J Clemens J Vaccine Protection of Bangladeshi infants and young children against cholera: implications for vaccine deployment and person-to-person transmission Pediatr Infect Dis J 2008 27 1 33 7 10.1097/INF.0b013e318149dffd 18162935

[B21] Kim D Canh DG Poulos C Thoa LTK Cook J Nguyen HT Nyamete A Dang TD Thuy MD Deen J Clemens J Thiem VD Anh DD Whittington D Private demand for cholera vaccines in Hue, Vietnam Value Health 2008 11 1 119 28 10.1111/j.1524-4733.2007.00220.x 18237366

[B22] Islam Z Maskery B Nyamete A Horowitz MS Yunus M Whittington D Private demand for cholera vaccines in rural Matlab, Bangladesh Health Policy 2008 85 2 184 95 10.1016/j.healthpol.2007.07.009 17822799

[B23] Lucas ME Jeuland M Deen J Lazaro N MacMahon M Nyamete A Barreto A von Seidlein L Cumbane A Songane FF Whittington D Private demand for cholera vaccines in Beira, Mozambique Vaccine 2007 25 14 2599 609 10.1016/j.vaccine.2006.12.027 17258844

[B24] WHO Cholera Vaccine Weekly epidemiological record 2009 81 50 526 29

[B25] Mahoney RT Krattiger A Clemens JD Curtiss R The introduction of new vaccines into developing countries. IV: Global Access Strategies Vaccine 2007 25 20 4003 11 10.1016/j.vaccine.2007.02.047 17363119

[B26] Amarasinghe A Wichmann O Margolis HS Mahoney RT Forecasting dengue vaccine demand in disease endemic and non-endemic countries Human Vaccines 2010 6 9 745 753 10.4161/hv.6.9.12587 PMC3056060 20930501

[B27] World Health Organization Guidelines for the clinical evaluation of dengue vaccines in endemic areas 2008 World Health Organization: Geneva 10.1016/j.vaccine.2008.05.058 18597906

[B28] Moran M Guzman J Ropars AL Illmer A The role of Product Development Partnerships in research and development for neglected diseases International Health 2010 2 114 122 10.1016/j.inhe.2010.04.002 24037470

[B29] Buse K Walt G Global public-private partnerships: part II- what are the health issues for global governance? Bulletin of the World Health Organization 2000 78 5 699 709 10859865 PMC2560757

[B30] Buse K Harmer A *Global health: Making partnerships work*, in Briefing Paper 2007 Overseas Development Institute: London

[B31] Elias C Polices and Practices to Advance Global Heatlh Technologies Report of the CSIS Global Health Policy Center 2009 Center for Strategic & International Studies: Washington, DC 16

[B32] Anonymous Integrating Intellectual Property Rights and Development Policy 2002 London: Commission on Intellectual Property Rights

[B33] Commission on Intellectual Property Rights Public Health: Innovation and Intellectual Property Rights 2006 World Health Organization: Geneva 228

[B34] Mahoney RT Krattiger A Clemens JD Curtiss R The introduction of new vaccines into developing countries IV: global access strategies Vaccine 2007 25 20 4003 11 10.1016/j.vaccine.2007.02.047 17363119

